# An Interpretation of Medicine to the Lay Public

**Published:** 1917-10-13

**Authors:** 


### HEALTH AND DISEASE.

### AN INTERPRETATION OF MEDICINE TO THE LAY PUBLIC.

The production for the general public of a book on Health has been the object of many ambitions, and some of these have attained a fair measure of success. When the design has also included a discourse on Disease a much more chastened record falls to be made. At the outset stands the difficulty of a medium of expression, for a technical vocabulary is obviously impossible and equivalents in the vernacular are not easy to compass. Not a few authors have fallen between the two stools and have gained neither the professional nor the popular critic. Then comes the danger of over-emphasis, as a result of which the lay reader becomes the victim of all the possibilities. The unqualified proposition comes next in order, and has all the dangers of the half-truth. At the other extreme is the refinement which seeks to be comprehensive and succeeds in the shape of a vague and hazy impression. A much lower level is reached by the writer who provides an easy guide to diagnosis and discusses treatment as a simple enterprise. To recognise all these possible failures is the first step to success, and the next step is a firm resolution to avoid them. But something more is necessary. Fullness of knowledge, of course, and equally, sincerity of purpose. Still further, while maintaining interest and engaging attention, the dignity of the subject demands a note of seriousness equivalent to the importance of the debate and a simplicity of statement suitable to a non-technical audience. The combination is obviously hard to achieve, as not a few mischances freely illustrate.

### An Educated Public Opinion Wanted.

All these considerations receive emphasis from the circumstances of the present hour and of the future which is close at hand. More than ever the general public is concerned with questions of health and disease, and with the relations of these both to personal welfare and national values. On the other hand, the medical profession recognises with increasing conviction the need for an educated public opinion if the larger possibilities of medicine are to be realised or even attempted. Co-operation is the order of the day, and a full and competent definition of these possibilities is a necessary preliminary to a joint enterprise in which the medical expert and the legislator and administrator must each take an essential part. There is much ignorance and much misunderstanding. The layman, even yet, is inclined to think that medicine is a body of mystery with some humbug. That it is merely common-sense reinforced by scientific and technical knowledge is a creed not widely spread. Such a creed must be established, and for its establishment a statement of the position is an essential, and the statement to be effective must have the qualities which are here defined.

### Dr. Roger Lee's Great Achievement.

To write of complex questions in serious, simple, and accurate terms is a hard undertaking, and needs a very special equipment. It is not to the amateur or the novice that such a task may be hopefully committed. Yet to ask it from the highly trained expert is a large demand, and not every expert has the necessary qualifications. Consent means the putting aside, at least for a time, of technical ambitions which must necessarily have a more appealing quality than popular exposition. A tribute of appreciation is therefore due to the authority who listens to this claim, and still more to the authority who meets it with success. Such a tribute must certainly be paid to Dr. Roger I. Lee, the well-known Professor of Hygiene in Harvard University, and the occasion which calls for it is the issue of his book on "Health and Disease: their Determining Factors."* It has fallen to our lot to read many books on allied subjects and with kindred aims, but never have we read one which so fully succeeds in a task that is admittedly severe, and which, as we have already indicated, urgently requires a competent discharge. The writer shirks no difficulty. He faces all the facts and never urges his propositions beyond what is justified by evidence. Where knowledge halts he will not advocate conclusions, and he has no short cuts to truth and safety. Yet his scheme is a most comprehensive one, and his teaching, where justification exists, has no shadow of doubt about it. Of exaggeration for the purpose of effect and emphasis he knows nothing, and the cheap and popular guide to medicine finds no place in his pages. The tone is restrained and serious and scientific, and the appeal throughout is to knowledge and to intelligence.

### The Highest Art.

A final and highly important qualification is the dignified and yet simple and lucid style in which the book is written. It is serious reading, yet it is easy reading, and the art of the writer never displays itself. We make no apology for emphasising this quality. It cannot have been attained save by a very clear perception of the great purpose of the book and by a resolute determination to make this purpose the dominating consideration. In this respect in particular we venture to congratulate Professor Lee and to assure him that his pages command our warm admiration and sincere gratitude. He comes forward at the right moment with a book which is a necessity of the existing position, and in so doing he has served well his day and generation. Both the medical profession and the general public are his debtors, for he has offered a substantial contribution to a cause in which they are equally interested—namely, the

* *Health and Disease: their Determining Factors.* By Roger I. Lee, M.D. Boston: Little, Brown and Co., 1917. Pp. 378 + xv.

preservation of health and the prevention of disease. His book is a presentation of medical knowledge in so far as this may be appreciated by the lay reader, and its substance as well as its diction is regulated by the desire to convey a clear, calm, commonsense impression both of the knowledge we possess and of the difficulties and hindrances which stand in the path of further progress. It is the truth about medicine stated in a fashion capable of comprehension by any intelligent man.

### The Layman's Friend and Guide.

The earlier chapters of Professor Lee's book deal with such subjects as "Heredity," "Food," "Air," "Exercise and Work," "Alcohol," and "Tobacco." All of these offer opportunities for sensationalism and dogmatism, and to this temptation not a few of our popular manuals have succumbed, with the result that the self-confident amateur, inspired by their pages, rushes in with opinions and advice where more angelic beings fear to tread. From provocations of this order, Dr. Lee, as was to be expected, keeps himself wholly free. The simple formula and the cheap prescription may appeal to the anxious inquirer and the popular lecturer, but they are at best only half-truths. Medical practitioners know well the harm they inflict, and how out of them come presumption and an unjustified self-confidence. No such results will arise from the study of Dr. Lee's book. On the contrary, the layman who makes it his study will be brought to recognise that there are relatively few propositions which stand secure, while many ready and repeated assertions are wholly without warrant.

### An Education in Scientific Method.

Much the same may be said of the chapters which deal with diseases, and the difficulties in presenting the true position here are obviously very great. To say that every detailed statement advanced by Dr. Lee will command universal medical approval would be going too far. The region is one where opinion on many points can only be tentative and where differences in the position and degree of emphasis must inevitably exist. But it can fairly be said that the information afforded has invariably an educative influence, and that while arousing interest and providing knowledge it is never calculated to provoke presumption or to stimulate dogmatism. Readers will recognise what medicine has accomplished, and the means by which these achievements have been secured. They will recognise also what severe conditions attend attempts to add to this knowledge, and what caution is necessary before claims to new truths can be allowed. In short, the difficulties and hesitations of the medical position are set forth not less than its triumphs and successes, and in this sense the book is an education in scientific method. The lay public must understand this method—its slowness as well as its successes—before medicine can command comprehension or co-operation, and it is because Professor Lee's book is a most effective aid to this end that we commend it to the attention and appreciation of all our readers.

